# Outlines of a New System of Physiology

**Published:** 1849-02

**Authors:** 


### OUTLINES OF A NEW SYSTEM OF PHYSIOLOGY.

Dr. Mackall, the author of this pamphlet, conceives that he has discovered certain general laws relating to physiology, which have heretofore escaped observation. We have not found time since we received his pamphlet to look into the grounds upon which his claims to discovery rest, but we must say that we have but little faith in physiological discoveries reasoned out in the closet. The author has not informed us that he has pursued any original researches for the advancement of physiology. In time, he proposes to lay before the world a full account of his views, of which the tract before us presents only an outline.

